# Integrating metagenomic next-generation sequencing into a multimodal diagnostic framework for spinal infection: enhancing etiological identification and clinical prediction

**DOI:** 10.3389/fcimb.2026.1904634

**Published:** 2026-08-24

**Authors:** Heng Liang, Hongyuan Qin, Jiayi Chen, Chengyu Qin, Xulin Li, Qiaojun Wang, Guoming Luo, Yuanming Chen

**Affiliations:** Department of Spinal Orthopedics, The Second Affiliated Hospital of Guangxi Medical University, University East Road, Nanning, Guangxi, China

**Keywords:** antibiotic exposure, diagnostic performance, metagenomic next-generation sequencing, pathogen identification, spinal infection

## Abstract

**Background:**

Spinal infection (SI) remains diagnostically challenging because of heterogeneous etiologies, nonspecific clinical manifestations, and the limited sensitivity of conventional microbiological approaches, particularly following empirical antimicrobial exposure. Although metagenomic next-generation sequencing (mNGS) enables unbiased pathogen detection, its incremental clinical value beyond pathogen identification and its role within integrated diagnostic strategies remain incompletely established.

**Methods:**

We retrospectively analyzed 208 consecutive patients with suspected SI between August 2022 and August 2025. Final diagnoses were established using a multidisciplinary-adjudicated composite reference standard incorporating clinical, radiological, microbiological, and histopathological evidence. The diagnostic performance of mNGS was compared with conventional culture and histopathology. Furthermore, multimodal predictive models integrating clinical variables and microbiological information were developed using L1-regularized logistic regression.

**Results:**

In the comparative cohort, mNGS achieved a significantly higher diagnostic yield than culture (66.5% vs. 27.41%, *P* < 0.001). Among confirmed SI cases, mNGS demonstrated higher sensitivity than conventional culture (91.67% vs. 40.15%, *P* < 0.001). mNGS identified a substantially broader pathogen spectrum, ranging from fastidious organisms such as *Mycobacterium tuberculosis* and *Brucella* to rare pathogens including *Talaromyces marneffei* and *Coxiella burnetii*, and maintained robust sensitivity (98.2%) despite prior antibiotic exposure. While an integrated clinical model achieved an AUC of 0.916, mNGS as a standalone modality provided superior discriminative power (AUC = 0.889) compared to histopathology (AUC = 0.836), the Conventional Biomarker Model (AUC = 0.742), and culture (AUC = 0.693).

**Conclusions:**

mNGS is a high-yield diagnostic tool for spinal infection, particularly in culture-negative and antibiotic-pretreated scenarios. Integrating mNGS into a multimodal clinical framework facilitates etiological clarity and precision antimicrobial therapy.

## Highlights

- Metagenomic next-generation sequencing enhances etiological diagnosis of spinal infections, particularly in culture-negative and antibiotic-exposed cases. Integrated with clinical, microbiological, and pathological evidence, mNGS provides actionable diagnostic information to support antimicrobial optimization and precision management.

## 1 Introduction

Spinal infection (SI) represents a heterogeneous spectrum of infectious disorders involving the vertebral bodies, intervertebral discs, spinal canal, and adjacent paraspinal soft tissues (Yokota and Tali, 2023; Zou et al., 2024). Despite advances in diagnostic imaging and antimicrobial therapy, SI remains associated with substantial morbidity due to delayed diagnosis, inappropriate antimicrobial selection, and irreversible structural damage (Xu et al., 2025). The increasing prevalence of aging populations, immunosuppressive conditions, metabolic disorders, and invasive spinal procedures has contributed to a rising clinical burden of SI worldwide (Kim et al., 2023). Clinically, SI may manifest as vertebral osteomyelitis, spondylodiscitis, epidural abscess, or paraspinal abscess, with etiological diversity ranging from common pyogenic bacteria to granulomatous infections caused by Mycobacterium tuberculosis or Brucella species, as well as less frequent fungal pathogens (Babic and Simpfendorfer, 2017; Tsantes et al., 2020).

Accurate etiological identification remains fundamental for individualized management of SI, as pathogen-directed therapy is closely associated with treatment efficacy and clinical outcomes (Zimmerli, 2026). However, establishing microbiological diagnoses remains challenging because currently available approaches have inherent limitations (Baryeh et al., 2022). Conventional microbial culture, although considered a reference method, demonstrates suboptimal sensitivity, particularly for slow-growing, fastidious, or paucibacillary organisms, and its diagnostic yield is substantially reduced after empirical antimicrobial exposure (Wang et al., 2023; Li et al., 2025). Histopathological examination provides valuable evidence of inflammatory patterns and granulomatous changes but may not always enable definitive pathogen identification (Bürger et al., 2020). In addition, targeted molecular assays, such as GeneXpert MTB/RIF for Mycobacterium tuberculosis detection, offer rapid and sensitive identification of predefined pathogens but are inherently restricted by their pathogen-specific design and limited ability to detect unexpected or mixed infections (Qi et al., 2022). Therefore, complementary diagnostic approaches capable of broad-spectrum pathogen detection are urgently needed for complex SI cases.

Metagenomic next-generation sequencing (mNGS) has emerged as a culture-independent molecular diagnostic approach that enables broad-spectrum detection of microbial nucleic acids directly from clinical specimens (Elbehiry and Abalkhail, 2025). Unlike targeted molecular assays that rely on predefined pathogen-specific sequences, mNGS allows simultaneous identification of diverse microorganisms, including bacteria, fungi, viruses, and parasites, without requiring prior assumptions regarding the causative pathogen (Liu et al., 2025). This unbiased detection capability provides potential advantages in diagnostically challenging infections characterized by low microbial burden, prior antimicrobial exposure, atypical pathogens, or polymicrobial etiologies (Gu et al., 2021; Rodino and Simner, 2024). Although mNGS has demonstrated clinical utility in respiratory, bloodstream, and central nervous system infections, its application in SI remains less well characterized, particularly regarding its integration into routine diagnostic pathways (Simner et al., 2018; Wilson et al., 2019; Duan et al., 2021).

Despite increasing interest in mNGS-based diagnostics, previous studies investigating SI have predominantly focused on pathogen detection rates or comparisons with conventional culture methods (Zhang et al., 2026). However, the clinical value of a diagnostic modality extends beyond pathogen identification alone, as therapeutic decision-making requires comprehensive interpretation of microbiological findings together with clinical presentation, inflammatory status, imaging characteristics, and pathological evidence (Li et al., 2019; Nishizawa et al., 2025). Moreover, the incremental contribution of mNGS within an integrated diagnostic framework remains insufficiently investigated, particularly in cases where conventional approaches are limited by antimicrobial exposure, fastidious organisms, or uncommon pathogens.

Therefore, this study aimed to systematically evaluate the clinical utility of mNGS in patients with suspected SI using a multidisciplinary-adjudicated composite reference standard. We first compared the diagnostic performance of mNGS with conventional microbiological and histopathological approaches. We further characterized its ability to identify diverse pathogens in diagnostically challenging scenarios, including culture-negative and antibiotic-exposed cases. Finally, we explored the incremental value of integrating mNGS-derived microbiological information with clinical variables to establish a multimodal diagnostic framework for improving etiological clarification and supporting individualized antimicrobial management.

## 2 Materials and methods

### 2.1 Study design

This retrospective cohort study was conducted at the Second Affiliated Hospital of Guangxi Medical University and included consecutive patients with suspected SI between August 2022 and August 2025. Final diagnoses were established according to a composite reference standard (CRS) based on multidisciplinary evaluation of clinical manifestations, laboratory parameters, radiological findings, microbiological results, and histopathological evidence. The diagnostic performance of mNGS was assessed using the CRS as the reference standard and was compared with conventional diagnostic approaches. The study protocol was approved by the institutional Ethics Committee (No. 2025-KYL [091]), and the requirement for informed consent was waived because of the retrospective study design.

### 2.2 Study population

Patients were eligible if they fulfilled the following criteria: (1) clinical suspicion of SI, defined as new or progressive spinal pain and/or neurological symptoms accompanied by fever, elevated inflammatory markers (ESR or CRP), and imaging evidence of spinal infection on MRI, CT, or radiography, including discitis, spondylitis, spondylodiscitis, endplate destruction, or paraspinal and epidural abscess formation (Berbari et al., 2015; Yin et al., 2025); and (2) availability of mNGS results and conventional microbiological culture and/or histopathological examination performed from the same clinical specimen. Patients were excluded if they had (1) suspected exogenous contamination affecting mNGS interpretation or (2) incomplete clinical information.

Clinical data were retrospectively extracted from electronic medical records, including demographic characteristics, laboratory parameters, radiological findings, sampling procedures, and specimen characteristics. Sampling approaches included percutaneous needle biopsy and open surgery, while collected specimens were categorized as osseous, soft tissue, or mixed tissue samples. Microbiological and histopathological assessments included smear microscopy (Gram staining and acid-fast staining), conventional culture, histopathological examination, and mNGS analysis.

### 2.3 Sample collection and microbiological culture

All patients underwent preprocedural imaging evaluation with computed tomography (CT) and/or magnetic resonance imaging (MRI) to identify the suspected sites of spinal involvement and guide specimen acquisition. Tissue specimens were subsequently obtained through C-arm fluoroscopy guided percutaneous needle biopsy or open surgical procedures according to clinical indications. Fresh specimens were immediately divided into separate portions and transferred under sterile conditions to the institutional microbiology and pathology laboratories for conventional microbiological culture and histopathological examination.

Conventional microbiological investigations included aerobic bacterial culture, anaerobic culture, fungal culture, and mycobacterial culture. In parallel, an additional specimen obtained from the same sampling procedure was collected for mNGS analysis. Samples designated for sequencing were placed in sterile, nuclease free containers and transported to the Center for Medical Genetics and Genomics within 24 hours for processing. All microbiological, histopathological, and sequencing analyses were performed within the institutional diagnostic facilities.

### 2.4 mNGS procedure

mNGS was performed using a standardized sequencing workflow. Total nucleic acids, including DNA and RNA, were extracted from clinical specimens (Yugo Zhizhi Technology, Beijing, China), and sequencing libraries were constructed with a target insert size of 330–350 bp. Library quality assessment was performed using Qubit 4.0 fluorometry and Agilent 2100 Bioanalyzer before sequencing. Qualified libraries were sequenced on the Illumina NextSeq CN500 platform using a single end 75 bp sequencing strategy. Bioinformatic processing was performed by converting raw BCL files into FASTQ format. Sequencing reads were quality controlled by removing adapter sequences and low-quality reads (Q value ≤10 or reads containing >10% ambiguous bases) using fastp version 0.24.0. Human host sequences were removed by alignment against the human reference genome (GRCh38) using BWA version 0.7.15. Remaining microbial reads were classified by comparison with a comprehensive microbial reference database derived from NCBI RefSeq and GenBank, covering a broad spectrum of bacteria, fungi, viruses, parasites, and other microorganisms.

Microbial identification was based on integrated interpretation of organism-specific sequencing reads, relative abundance metrics such as reads per million (RPM), negative control results, specimen characteristics, and clinical compatibility. To minimize potential contamination, sterile no-template controls were processed concurrently with clinical samples, and all detected microorganisms were reviewed by experienced clinicians and laboratory specialists before being considered clinically relevant.

### 2.5 Definitions and outcome measures

Diagnostic Reference Standard: The final diagnosis of SI was determined according to a CRS based on multidisciplinary evaluation of clinical manifestations, laboratory parameters, radiological findings, microbiological results, histopathological evidence, and therapeutic response when appropriate. SI was considered confirmed when compatible clinical and imaging features were supported by microbiological evidence (culture or mNGS) and/or histopathological findings consistent with infection. For cases with discordant diagnostic results, particularly those involving fastidious or difficult-to-culture pathogens, sustained clinical improvement following targeted antimicrobial therapy, accompanied by normalization or substantial reduction of inflammatory markers (ESR and CRP) during a follow-up period of at least 3 months, was considered supportive evidence for final diagnostic adjudication.

Lesional Distribution and Extent: The anatomical distribution and extent of spinal involvement were recorded based on imaging findings. Multilevel involvement was defined as active infectious lesions affecting two or more spinal regions (cervical, thoracic, lumbar, or sacral), whether contiguous or non-contiguous. Multi-segmental involvement was defined as infectious involvement extending across three or more vertebral segments.

Histopathological interpretation: Histopathological positivity was defined as the presence of pathological changes compatible with infection, including acute or chronic inflammatory infiltration, granulomatous inflammation, necrotizing changes, tissue or bone destruction, and/or direct visualization of microorganisms.

Clinical outcomes: Clinical outcomes were recorded as supplementary clinical information during follow-up. Treatment response was categorized as improvement or failure. Improvement was defined as resolution of clinical symptoms accompanied by a ≥25% reduction in ESR or CRP from baseline (or ESR <40 mm/h and CRP <10 mg/L when baseline values were unavailable). Treatment failure was defined as the occurrence of infection-related death, microbiological recurrence, or mechanical failure requiring unplanned surgical intervention due to structural complications despite negative intraoperative microbiological findings (Gupta et al., 2014; Matsuo et al., 2026).

### 2.6 Statistical analysis

Continuous variables were expressed as mean ± standard deviation (SD) or median with interquartile range (IQR), according to data distribution, whereas categorical variables were summarized as frequencies and percentages. Comparisons between groups were performed using the independent samples t-test or Mann–Whitney U test for continuous variables and the chi-square test or Fisher’s exact test for categorical variables, as appropriate. The diagnostic performance of mNGS and conventional culture was evaluated using the CRS as the reference standard. Diagnostic yield, sensitivity, specificity, positive predictive value (PPV), and negative predictive value (NPV), together with their corresponding 95% confidence intervals (CIs), were calculated. Agreement between mNGS, conventional culture, and histopathological examination was assessed using Cohen’s kappa coefficient. To establish integrated diagnostic models for discriminating SI from non-SI, L1-penalized logistic regression was applied for variable selection and model construction, aiming to reduce overfitting and account for potential multicollinearity among candidate predictors. The discriminative performance of the developed models was evaluated using receiver operating characteristic (ROC) curve analysis and quantified by the area under the curve (AUC) with corresponding 95% CIs. All statistical analyses were performed using SPSS version 26.0 (IBM Corp., Armonk, NY, USA), GraphPad Prism version 9.5, and Python version 3.12.7. A two-sided P value <0.05 was considered statistically significant.

## 3 Results

### 3.1 Patient characteristics and diagnostic cohort

A total of 208 patients with suspected SI were included according to the predefined eligibility criteria and subsequently classified into the SI group (n = 138) and non-SI group (n = 70) based on the CRS. Among patients with confirmed SI, 73 were male and 65 were female, with a median age of 62 years (IQR, 16 years). The lumbar spine was the most frequently affected region (n = 72, 52.2%). Baseline comparisons demonstrated significant differences between the SI and non-SI groups in erythrocyte sedimentation rate (ESR), C-reactive protein (CRP), D-dimer levels, and the number of involved spinal segments (all P < 0.05; **Table 1**).

**Table 1:** Demographic and clinical characteristics of the SI and non-SI groups.

Variable	SI group (N = 138)	Non-SI group (n=70)	P-value
Age (years), median (IQR)	62.00 (16)	66.00(19.75)	0.120
Gender, n (%)			0.405
Female	65 (47.1)	38 (54.3)	
Male	73 (52.9)	32 (45.7)	
Underlying disease, n (%)			
hypertension	37 (62.7)	19 (59.4)	0.931
diabetes	24 (40.7)	8 (25.0)	0.206
Osteoporosis, n (%)			0.005
No	73 (58.4)	25 (36.2)	
Yes	52 (41.6)	44 (63.8)	
Surgical history, n (%)			0.475
No	107 (77.5)	58 (82.9)	
Yes	31 (22.5)	12 (17.1)	
Lesional site, n (%)			0.167
Cervical	4 (2.9)	5 (7.1)	
Thoracic	24 (17.4)	16 (22.9)	
Lumbar	72 (52.2)	36 (51.4)	
Sacrum	2 (1.4)	0 (0.0)	
Paraspinal tissues	8 (5.8)	0 (0.0)	
Multilevel*	28 (20.3)	13 (18.6)	
Involved segments, n (%)			< 0.001
Single-segment	8 (6.3)	35 (53.8)	
Bi-segment	80 (63.0)	14 (21.5)	
Multi-segment^#^	39 (30.7)	16 (24.6)	
WBC(×10^^9^/L), median (IQR)	9.16 (7.17)	7.38 (3.58)	0.015
PLT(×10^^12^/L), median (IQR)	316.00 (146.00)	290.00 (96.00)	0.010
N (%), median (IQR)	0.76 (0.15)	0.67 (0.15)	< 0.001
L (%), median (IQR)	0.15 (0.13)	0.21 (0.12)	< 0.001
MONO (%), median (IQR)	0.06 (0.03)	0.07 (0.02)	0.286
ESR (mm/h), median (IQR)	84.00 (52.50)	42.50 (37.00)	< 0.001
CRP (mg/L), median (IQR)	46.30 (96.85)	11.25 (45.47)	< 0.001
D-dimer (ng/ml), median (IQR)	595.00 (568.50)	415.00 (545.00)	0.023

*Multilevel was defined as involvement of two or more distinct anatomical regions of the spine (i.e., cervical, thoracic, lumbar, or sacral). ^#^Multi-segment was defined as lesions extending across three or more vertebral segments. SI, spinal infection; WBC, white blood cell count; PLT, platelet count; N, Neutrophil; L, lymphocyte; MONO, Monocyte; ESR, erythrocyte sedimentation rate; CRP, C-reactive protein.

Of the 208 enrolled patients, 11 were excluded from the comparative analysis of mNGS and conventional culture because corresponding culture results were unavailable. Therefore, diagnostic performance comparisons were conducted in the matched microbiological cohort.

### 3.2 Diagnostic yield and pathogen spectrum of mNGS compared with conventional culture

To evaluate the diagnostic contribution of mNGS in suspected SI, pathogen detection profiles were first compared between mNGS and conventional culture in the matched cohort (N = 197). mNGS identified clinically relevant microorganisms in 131 cases (66.5%), whereas conventional culture yielded positive results in 54 cases (27.4%). Among patients with confirmed SI and available culture results (n = 132), mNGS demonstrated a substantially higher detection rate than conventional culture (91.7% [121/132] vs. 40.2% [53/132], P < 0.001). In the entire SI cohort (n = 138), mNGS identified pathogens in 127 patients (92.0%), supporting its consistent diagnostic capability across the complete clinical population.

**Figure 1:**
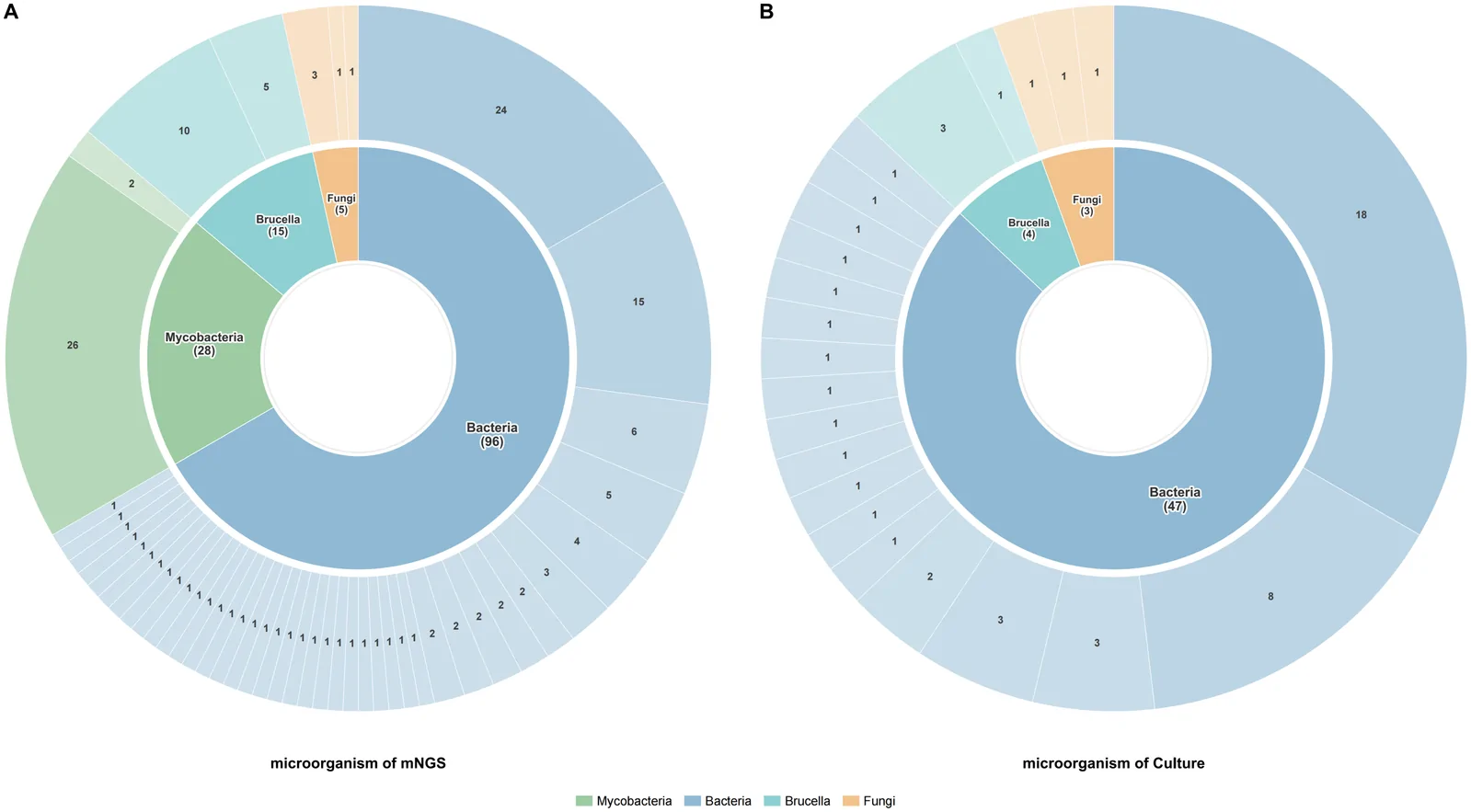
Comparison of microbial profiles identified by mNGS and conventional culture in spinal infection. **(A)** mNGS; **(B)** conventional culture. Inner rings denote major microbial categories, and outer rings depict species-level pathogen distribution with corresponding detection counts.

**Figure 2:**
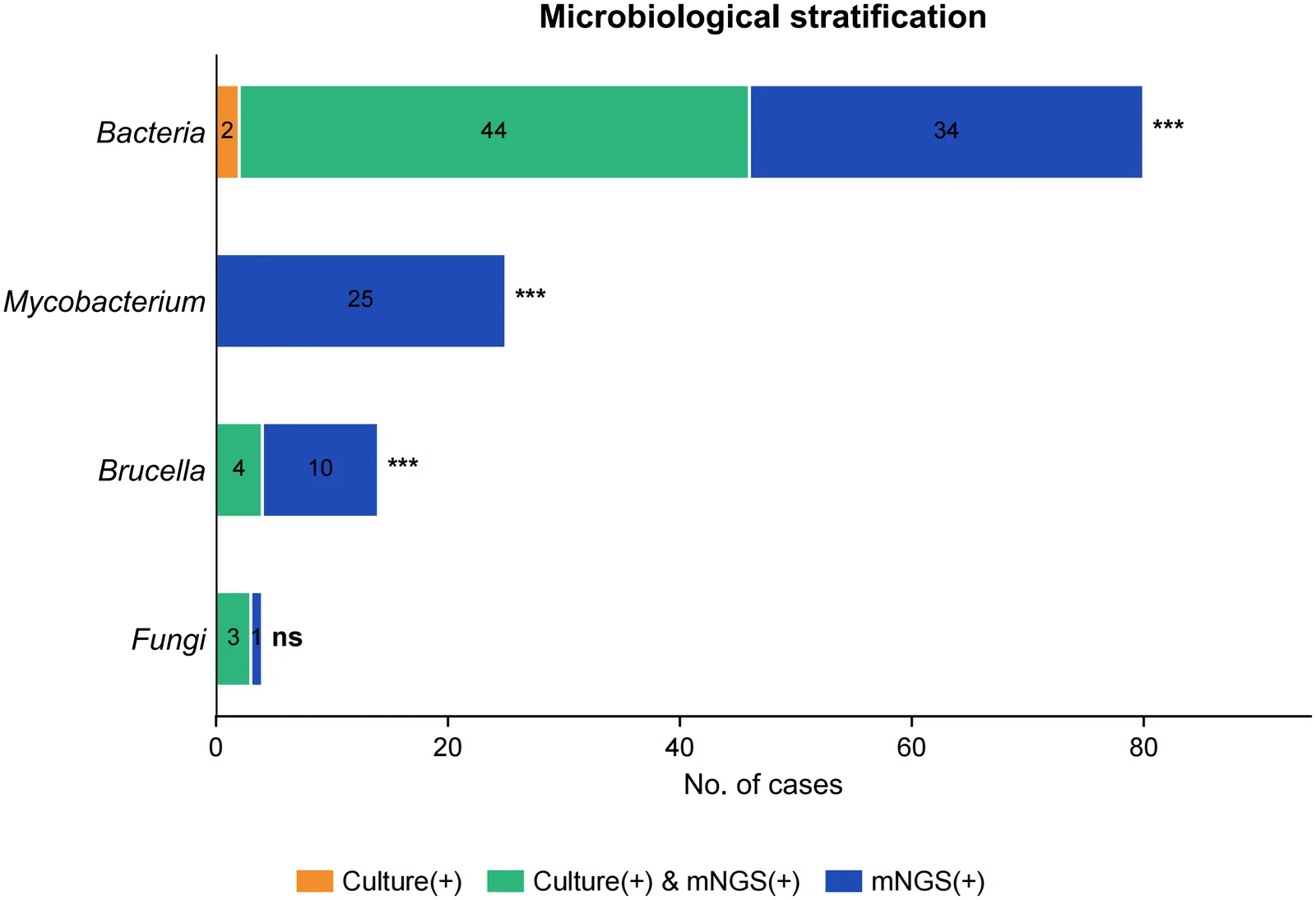
Comparison of diagnostic yield between mNGS and conventional culture across different pathogen categories. *** indicates P < 0.001; ns indicates no statistically significant difference.

The pathogen distribution identified by the two modalities is illustrated in **Figures 1**, **2**. Conventional culture predominantly detected common bacterial pathogens, including *Staphylococcus aureus* (n = 18), *Escherichia coli* (n = 8), *Brucella species* (n = 4), *Klebsiella pneumoniae* (n = 3), and *Salmonella enterica* (n = 3). In contrast, mNGS revealed a broader microbial spectrum, with *Mycobacterium tuberculosis complex* (n = 26), *Staphylococcus aureus* (n = 24), *Escherichia coli* (n = 15), and *Brucella species* (n = 10) being the most frequently identified organisms. Pathogen-specific comparison demonstrated that mNGS provided additional diagnostic information, particularly for fastidious organisms. Compared with culture, mNGS identified significantly more cases of *Mycobacterium species* (25 vs. 0, P < 0.001) and *Brucella species* (14 vs. 4, P < 0.001). No significant difference was observed between the two modalities for fungal detection.

### 3.3 Concordance and complementary diagnostic value between mNGS and conventional culture

**Figure 3:**
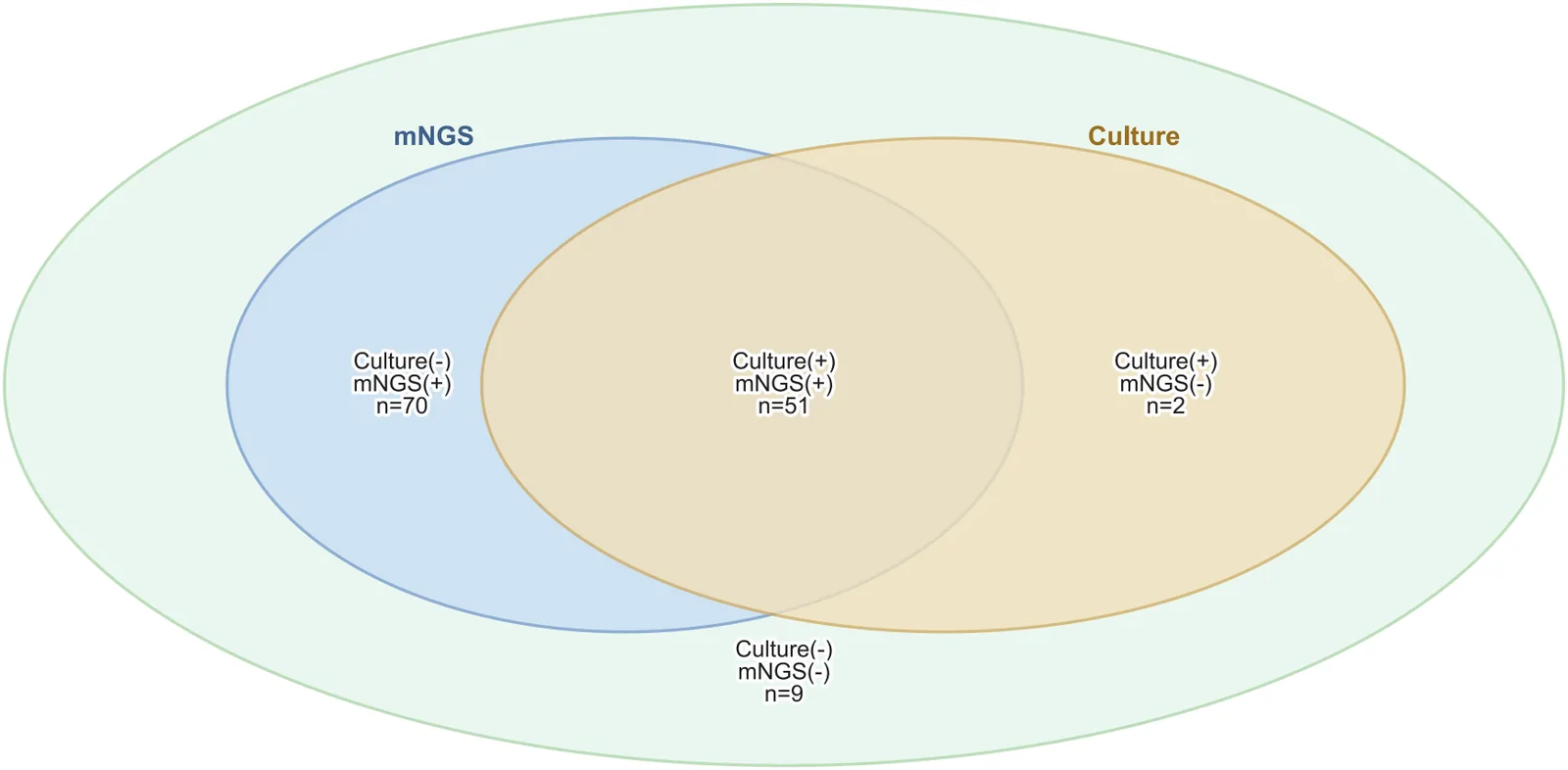
Diagnostic yield and overlap of mNGS versus conventional culture.

**Figure 4:**
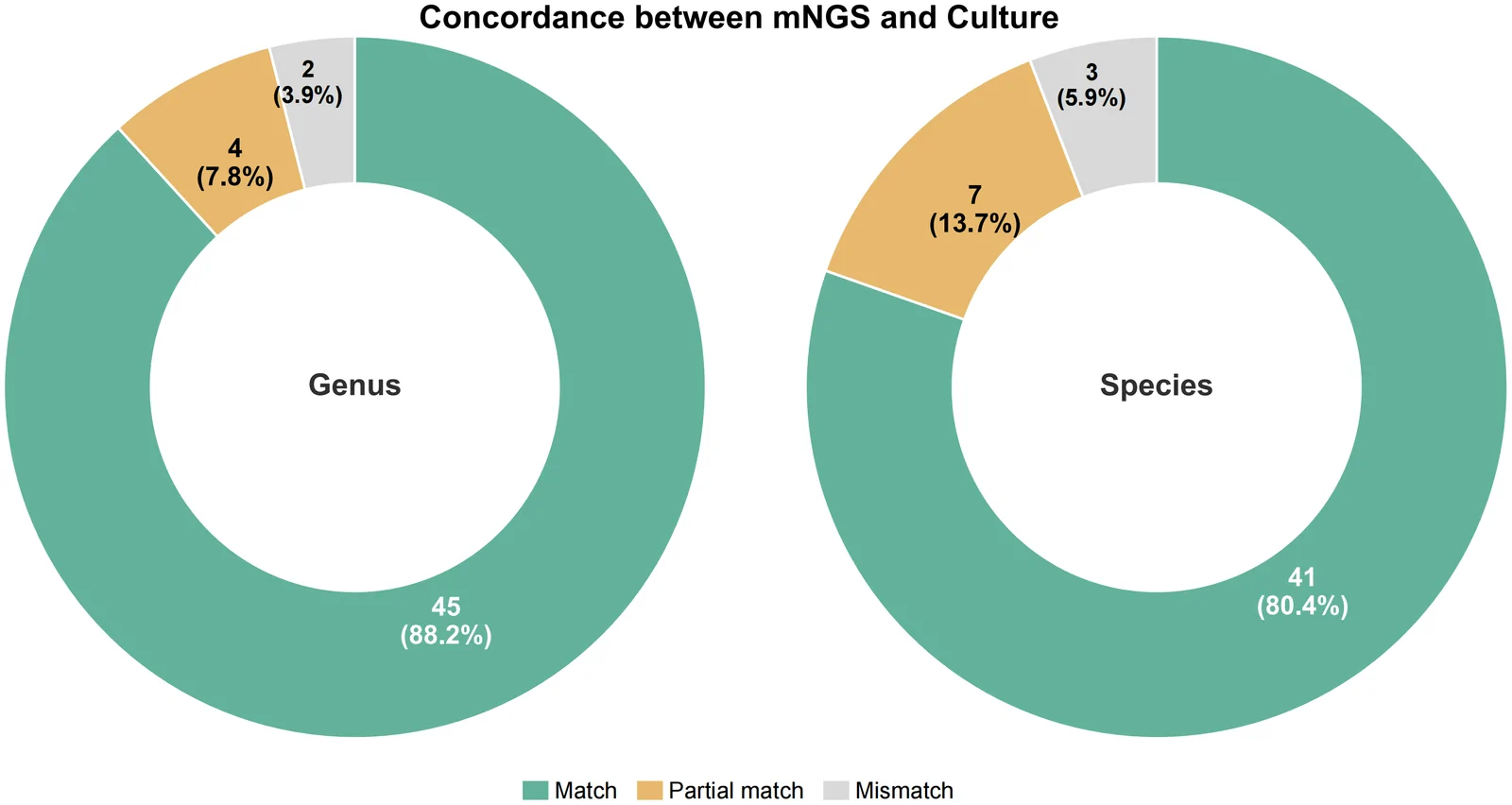
Concordance of pathogen identification between mNGS and conventional culture at the genus and species levels. Match: Both methods identified the same pathogen. Partial match: One method identified multiple pathogens, including the one found by the other method, or identification matched only at the genus level. Mismatch: The pathogens identified by the two methods were entirely different.

The agreement and complementary characteristics of mNGS and conventional culture were further assessed in patients with confirmed SI (**Figure 3**). Concordant positive results were observed in 51 cases (38.6%), whereas both methods yielded negative results in 9 cases (6.8%). Among the dual-positive cases, mNGS and culture demonstrated high taxonomic concordance, with genus-level and species-level agreement rates of 88.2% (45/51) and 80.4% (41/51), respectively (**Figure 4**; **Supplementary Figure 1**).

**Figure 5:**
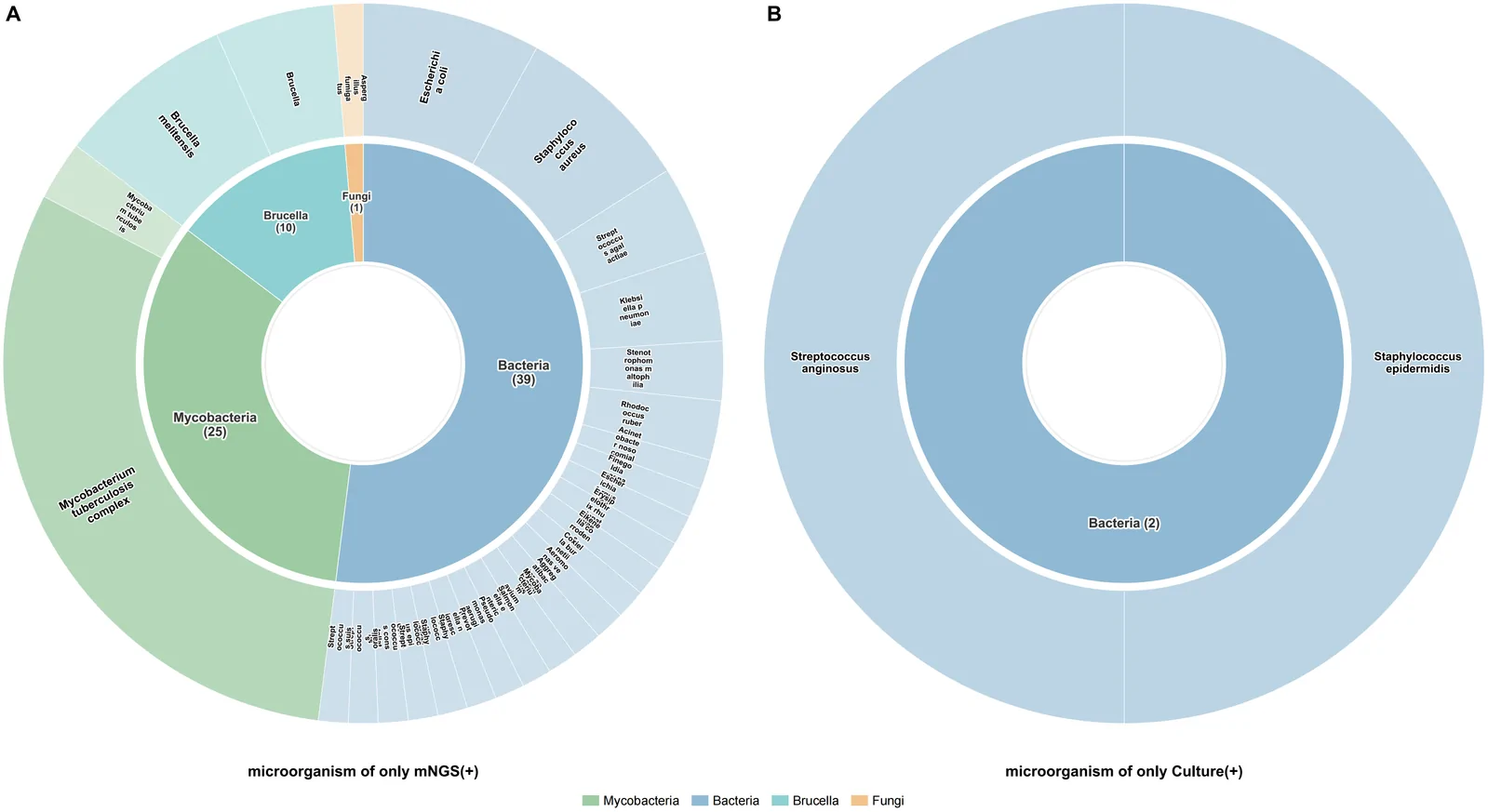
Distribution of microorganisms detected exclusively by mNGS **(A)** or conventional culture **(B)**. The inner ring represents the category of the microorganism, and the outer ring represents the specific species.

Discrepancy analysis further demonstrated the complementary diagnostic value of mNGS. A total of 74 additional microorganisms were identified exclusively by mNGS among 70 culture-negative patients (**Figure 5A**), with *Mycobacterium tuberculosis complex* being the most frequently detected organism (n = 23). In addition, mNGS enabled identification of several fastidious or uncommon pathogens that are difficult to recover using conventional culture, including *Aggregatibacter aphrophilus, Finegoldia magna*, and *Coxiella burnetii*. Conversely, only two cases were positive exclusively by culture, involving *Staphylococcus epidermidis* and *Streptococcus anginosus* (**Figure 5B**).

### 3.4 Robustness of mNGS performance across antibiotic exposure and sampling characteristics

**Figure 6:**
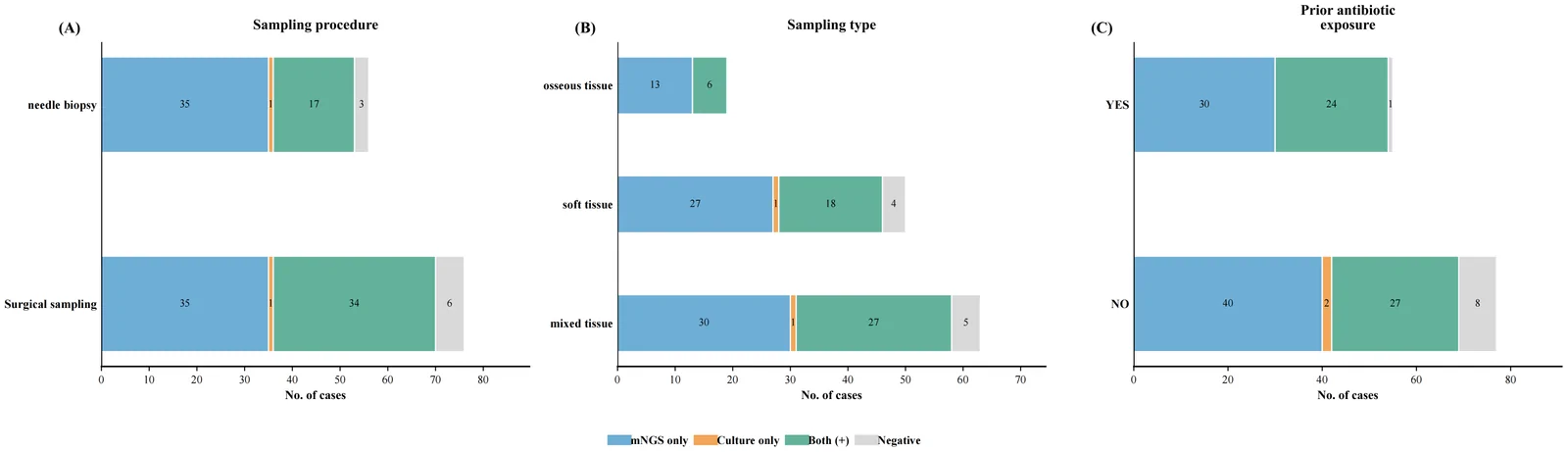
Impact of clinical variables on the distribution of pathogen detection results between mNGS and conventional culture. **(A–C)** Detection patterns stratified by sampling procedure, specimen type, and prior antibiotic exposure, respectively. Detection outcomes included mNGS-positive only, culture-positive only, dual positivity, and negative results.

To evaluate whether clinical and procedural factors influenced mNGS performance, subgroup analyses were performed according to sampling approach, specimen type, and prior antibiotic exposure (**Figure 6**). The distribution of concordant detection and mNGS-exclusive identification was comparable between percutaneous biopsy and surgical specimens, with mNGS identifying additional pathogens in 35 percutaneous biopsy samples and 35 surgical specimens (**Figure 6A**). Similar patterns were observed across different specimen categories, including osseous, soft tissue, and mixed tissue samples (**Figure 6B**). Among the 55 patients with prior antibiotic exposure before specimen acquisition, mNGS maintained a high diagnostic capability, with mNGS-exclusive and concordant detection rates of 54.5% and 43.6%, respectively (**Figure 6C**). Neither sampling approach, specimen composition, nor antecedent antibiotic exposure significantly altered the distribution of diagnostic outcomes (all P > 0.05).

**Figure 7:**
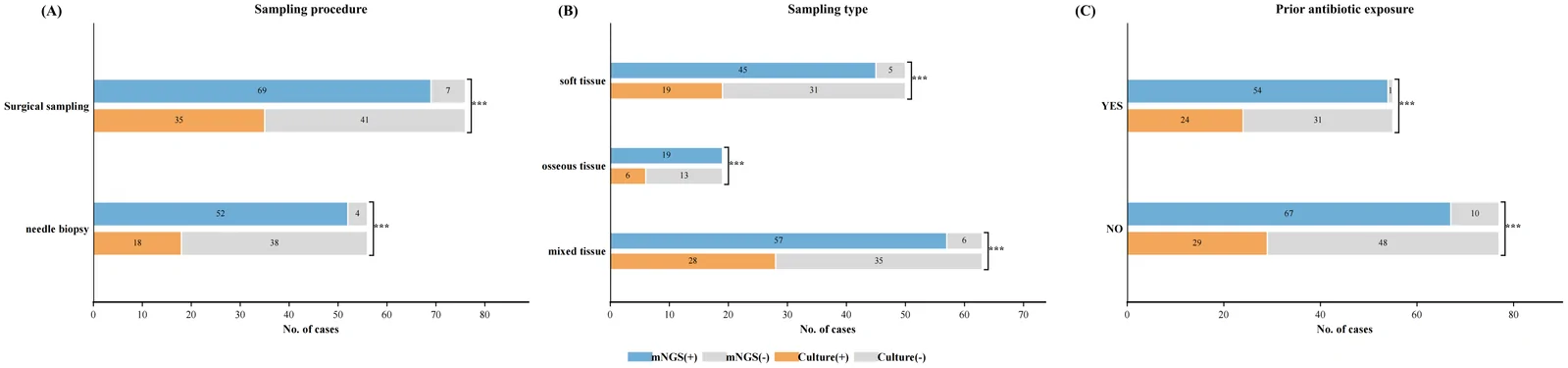
Comparative analysis of pathogen detection rates between mNGS and conventional culture across clinical subgroups. **(A–C)** Pathogen detection rates of mNGS and conventional culture were compared according to sampling procedures **(A)**, sampling types **(B)**, and prior antibiotic exposure status **(C)**. Within each clinical subgroup, mNGS demonstrated significantly higher detection rates than conventional culture. Between-subgroup comparisons showed no significant differences in detection performance for either mNGS or culture across different sampling strategies, specimen categories, or antibiotic exposure conditions. *** indicates P < 0.001.

Subgroup analyses further demonstrated that mNGS consistently achieved higher sensitivity than conventional culture across all clinical strata (all P < 0.001; **Figure 7**). The sensitivity of mNGS remained stable across different sampling approaches (90.8%–92.9%), specimen categories (90.0%–100.0%), and antibiotic exposure status (87.0%–98.2%). These findings indicate that the diagnostic performance of mNGS was maintained across diverse real-world clinical scenarios.

### 3.5 Antimicrobial resistance profiling by mNGS

**Figure 8:**
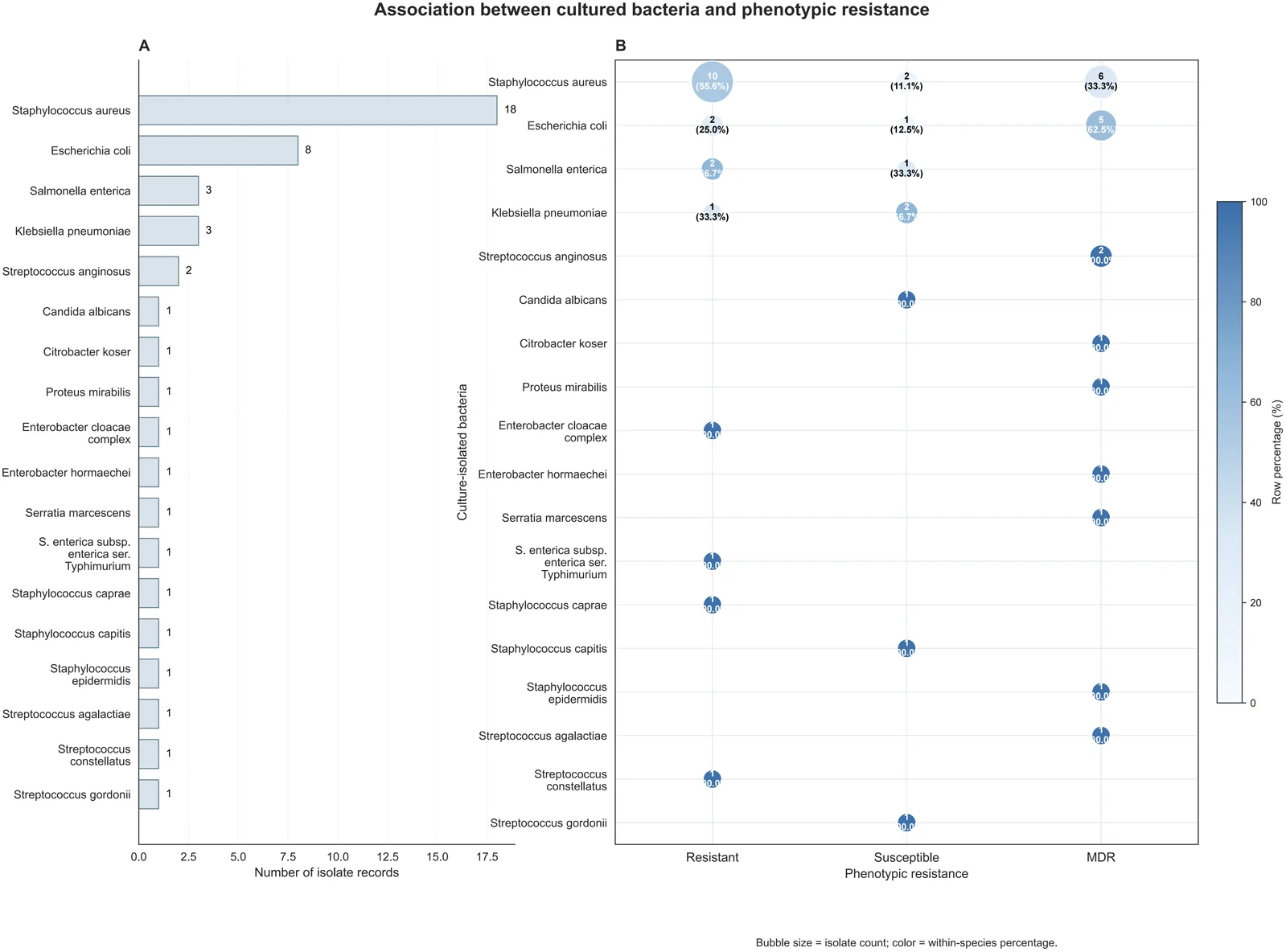
Isolated bacterial species and corresponding phenotypic resistance. **(A)** Frequency of isolated pathogens. **(B)** Distribution of resistance categories, including multidrug resistance (MDR), across species.

The relationship between mNGS-derived antimicrobial resistance gene (ARG) profiles and phenotypic antimicrobial susceptibility was evaluated among culture-positive cases with available antimicrobial susceptibility testing results. Among the 53 culture-positive specimens, *Staphylococcus aureus* (n = 18) and *Escherichia coli* (n = 8) were the predominant organisms available for resistance analysis. Phenotypic multidrug resistance was identified in 62.5% (5/8) of *Escherichia coli* isolates and 33.3% (6/18) of *Staphylococcus aureus* isolates. Overall, 88.9% (16/18) of *Staphylococcus aureus* isolates exhibited resistance to at least one antimicrobial category (**Figure 8**).

**Figure 9:**
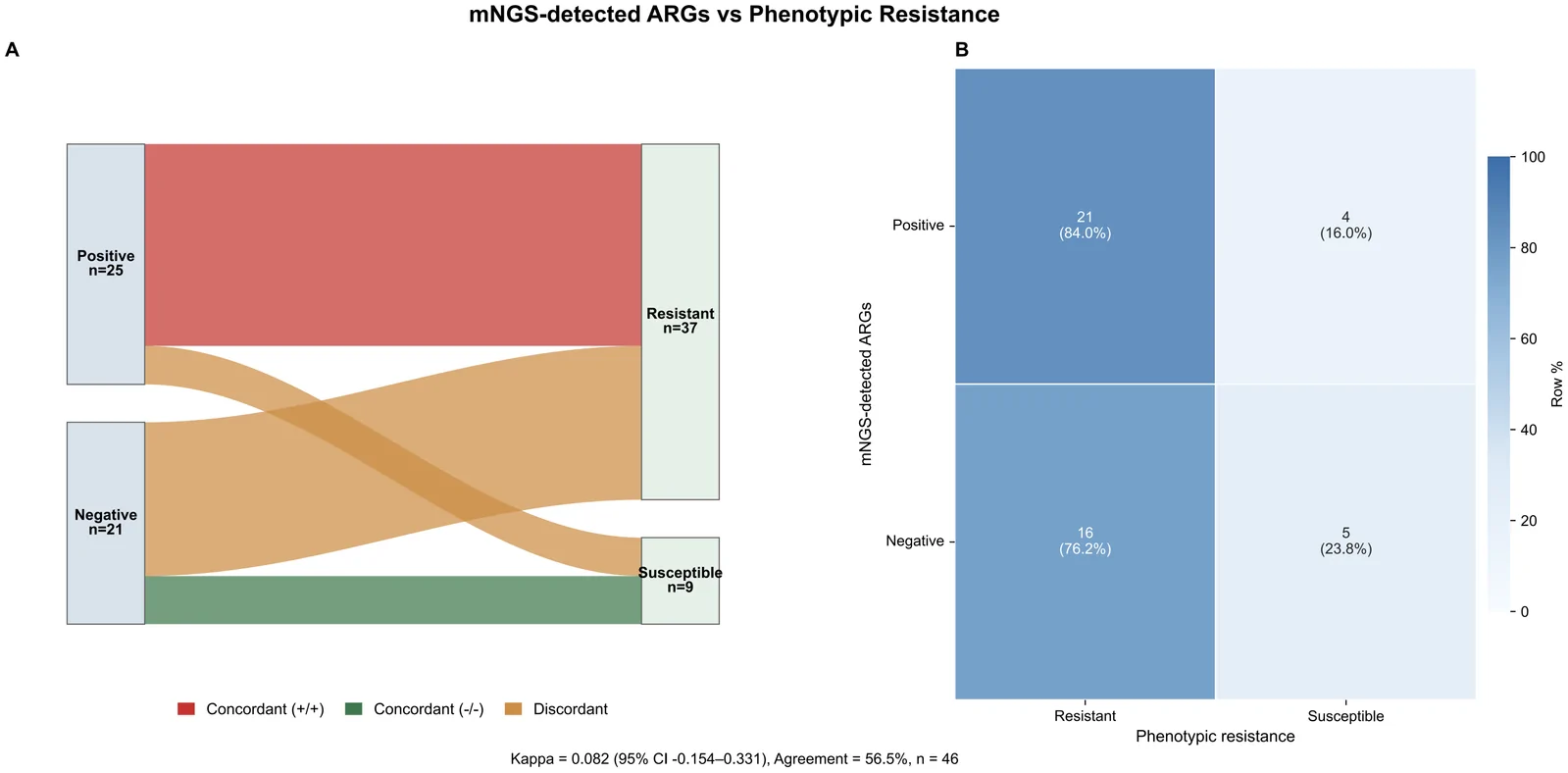
Agreement between mNGS-detected ARGs and phenotypic resistance. **(A)** Sankey diagram illustrating the flow of concordance and discordance. **(B)** Heatmap showing the correlation between genotypic and phenotypic profiles.

Among the 46 cases with available antimicrobial susceptibility testing results, mNGS-derived ARG profiles showed a sensitivity of 56.8% (21/37), specificity of 55.6% (5/9), and positive predictive value of 84.0% (21/25) for corresponding phenotypic resistance patterns. However, the correlation between ARG profiles and phenotypic resistance was weak, with a Cohen’s kappa coefficient of 0.082. These findings indicate that ARG information obtained from mNGS may provide complementary genomic evidence regarding antimicrobial resistance but should not be interpreted as a replacement for conventional phenotypic susceptibility testing (**Figure 9**).

**Figure 10:**
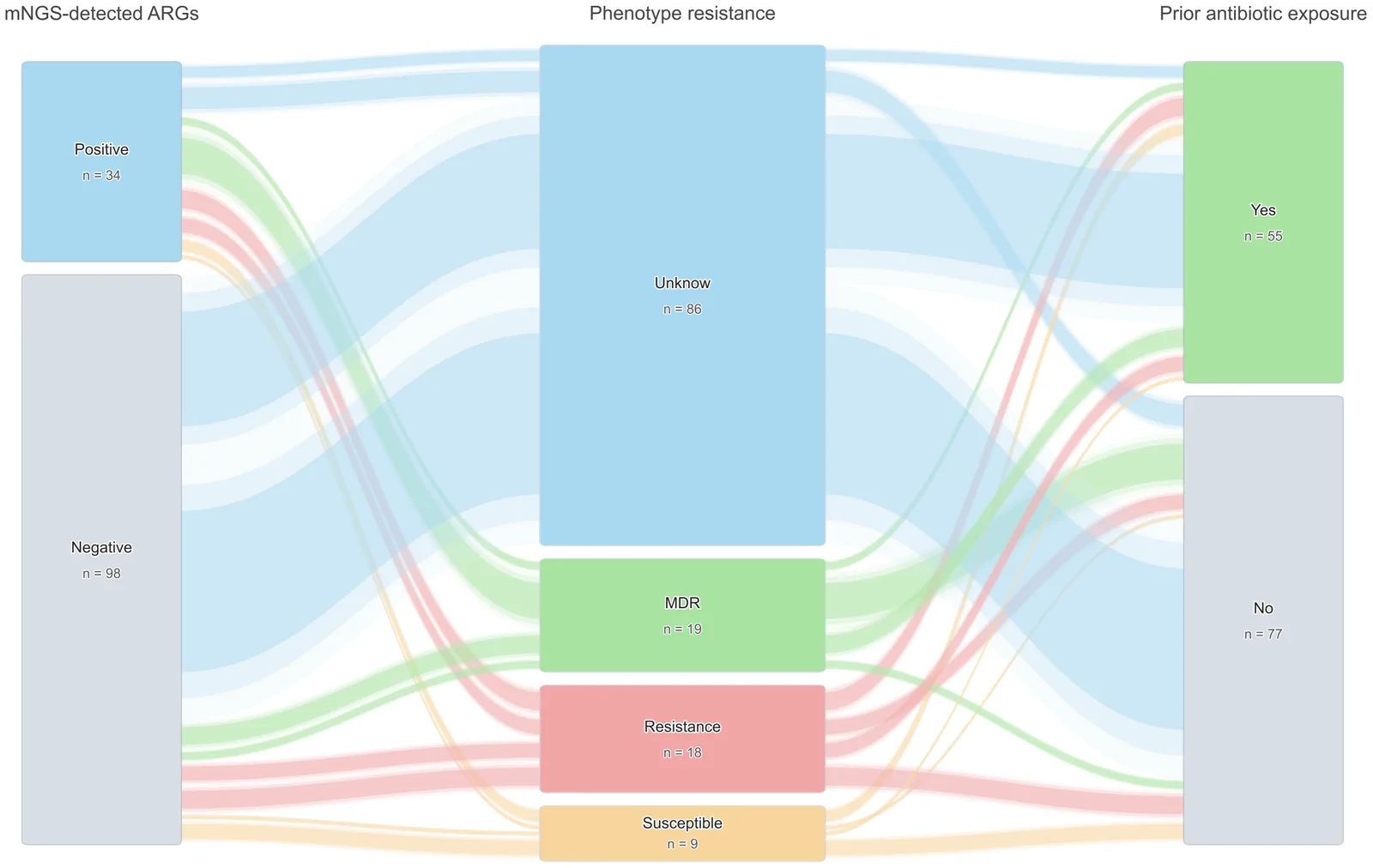
Interplay between mNGS-detected ARGs, phenotypic resistance, and prior antibiotic exposure (N = 132).

In the overall SI cohort, ARGs were identified by mNGS in 34 patients, including individuals without positive culture results (**Figure 10**). ARG detection was significantly less frequent among patients without available phenotypic susceptibility data, most of whom represented culture-negative cases, compared with those with corresponding susceptibility results (10.5% [9/86] vs. 54.3% [25/46], P < 0.001). Prior antibiotic exposure was not significantly associated with ARG detection (25.3% vs. 23.6%, P = 0.824) or phenotypic antimicrobial resistance patterns (80.8% vs. 80.0%, P = 1.000) (**Supplementary Table 1**).

### 3.6 Comparative diagnostic performance and integrated diagnostic modeling

**Figure 11:**
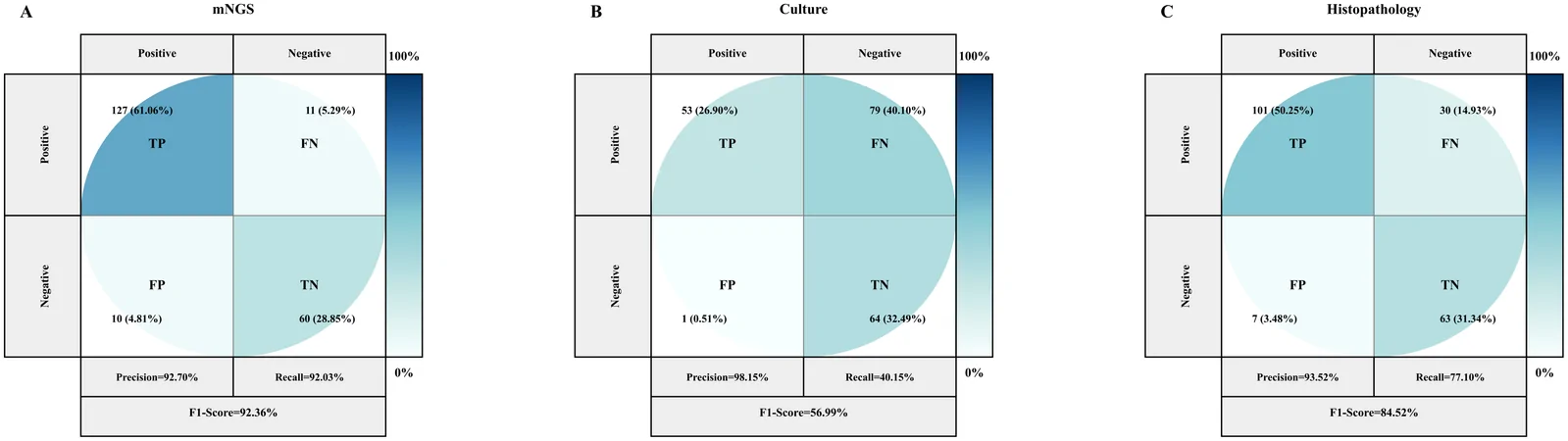
Confusion matrices comparing the diagnostic performance of mNGS **(A)**, conventional culture **(B)**, and histopathology **(C)**.

The diagnostic performance of mNGS, conventional culture, histopathology, and integrated diagnostic models was further evaluated using the CRS as the reference standard (**Figure 11**). In the complete cohort, mNGS demonstrated a sensitivity of 92.0% (95% CI: 86.3%–95.5%) and an overall accuracy of 89.9%, exceeding those observed for histopathology (77.1% sensitivity and 81.6% accuracy) and conventional culture (40.2% sensitivity and 59.4% accuracy) (**Supplementary Tables 2, S3**). Similar results were observed in the matched cohort (N = 190), where mNGS achieved a sensitivity of 91.2% and accuracy of 88.9% (**Supplementary Table 4**). The detection agreement among diagnostic modalities was assessed using Cohen’s kappa analysis. Agreement was moderate between mNGS and histopathology (κ = 0.41), whereas lower agreement was observed between mNGS and culture (κ = 0.27) and between culture and histopathology (κ = 0.18) (**Supplementary Table 5**).

**Figure 12:**
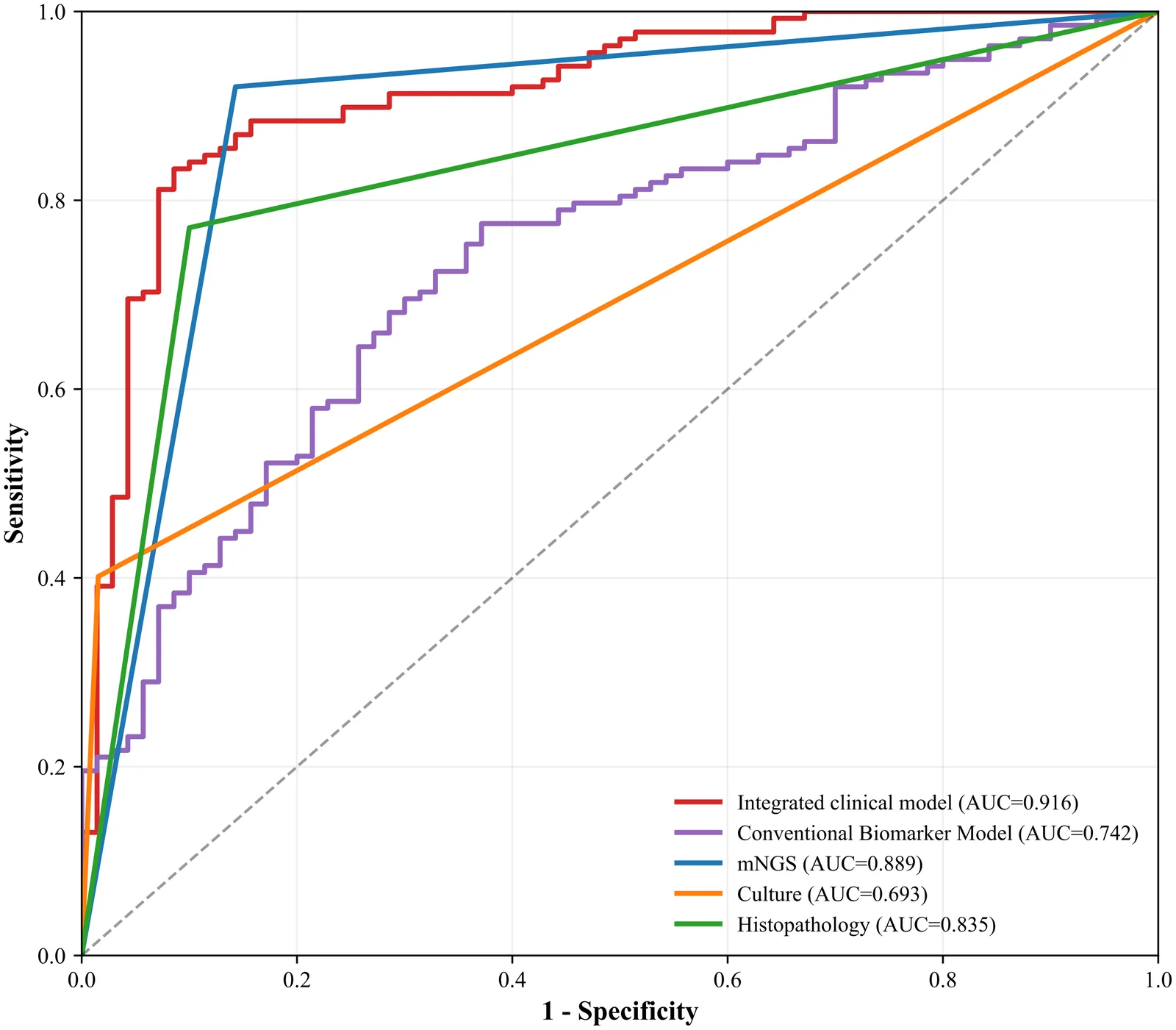
ROC curves comparing the diagnostic performance of microbiological tests, Histopathology, and the two predictive models. AUC, area under the curve. The Integrated clinical model (incorporating clinical, imaging, and laboratory features) and the Conventional biomarker model (based on laboratory blood markers) were developed using L1-regularized logistic regression. 95% confidence intervals for AUC were estimated using the bootstrap method (n=1000).

ROC curve analysis was subsequently performed to evaluate the discriminatory capability of individual modalities and integrated diagnostic models for distinguishing SI from non-SI (**Figure 12**; **Supplementary Figure 2, S3**). The integrated clinical model demonstrated the highest diagnostic discrimination, with an AUC of 0.916 (95% CI: 0.870–0.952). Among individual diagnostic modalities, mNGS showed the strongest discriminatory performance (AUC = 0.889), followed by histopathology (AUC = 0.836), the conventional biomarker model (AUC = 0.742), and culture (AUC = 0.693) (**Supplementary Tables 6–S8**).

### 3.7 Clinical implications of etiological identification and treatment outcomes

Clinical management strategies and follow-up outcomes were evaluated to further characterize the clinical implications of etiological identification in patients with CRS-confirmed SI. The median length of hospitalization was 18 days (IQR, 13–25.3 days), and the median follow-up duration was 73 days (IQR, 31–165.5 days). Among the 138 patients with confirmed SI, 119 (86.2%) underwent surgical intervention, whereas 19 (13.8%) received conservative management (**Supplementary Table 9**).

Antimicrobial regimens were tailored according to the final etiological diagnosis, incorporating microbiological findings from mNGS and conventional culture when available, together with pathological and clinical assessments. Pyogenic spinal infections were primarily managed with pathogen-directed antibacterial therapy, whereas tuberculous spondylitis was treated with standard multidrug anti-tuberculosis regimens, brucellar spondylitis with combination antimicrobial therapy, and fungal infections with species-specific antifungal treatment (**Supplementary Table 10**). Following etiological clarification, antimicrobial regimens were modified in 112 of 138 patients (81.2%). Specifically, 108 patients (78.3%) received targeted antimicrobial therapy, including 44 patients (31.9%) who were treated for specific infectious entities requiring pathogen-directed regimens, such as tuberculous or brucellar spondylitis. Antibiotic de-escalation was performed in four patients (2.9%).

At hospital discharge, 134 patients (97.1%) demonstrated clinical improvement, whereas three patients (2.2%) experienced clinical progression and one patient (0.7%) died. During follow-up, treatment success was achieved in 125 patients (90.6%). Treatment failure occurred in 13 patients (9.4%), including infection-related mortality (n = 7, 5.1%), microbiological recurrence (n = 4, 2.9%), and mechanical failure requiring additional intervention (n = 2, 1.4%) (**Supplementary Table 9**).

## 4 Discussion

In this study, we comprehensively evaluated the diagnostic value of mNGS in patients with suspected SI using a multidisciplinary composite reference standard and investigated its contribution within an integrated diagnostic framework. Rather than viewing mNGS as a standalone diagnostic modality, we evaluated its incremental contribution to etiological clarification through integration with conventional microbiological, histopathological, and clinical evidence. Our findings indicate that the principal value of mNGS lies not merely in increasing pathogen detection, but in enhancing etiological resolution in clinically challenging scenarios involving culture-negative infections, prior antimicrobial exposure, and fastidious or uncommon pathogens (Lv et al., 2024; Shi et al., 2024). Moreover, integrating mNGS-derived pathogen information with clinical parameters enhanced diagnostic discrimination between SI and non-SI, supporting the potential role of mNGS as a complementary component of a multimodal diagnostic framework for spinal infection.

The most prominent advantage of mNGS was observed in diagnostically challenging infections where conventional approaches are frequently limited. In our cohort, mNGS identified pathogens in 69 culture-negative cases, highlighting its complementary value when standard microbiological methods fail (Xu et al., 2022; Chen et al., 2024). This benefit was particularly evident in spinal tuberculosis, a typical paucibacillary infection characterized by low organism burden and prolonged culture requirements (Huang et al., 2023). Conventional culture and acid-fast bacilli staining showed limited sensitivity in these cases, whereas mNGS detected Mycobacterium tuberculosis complex in 29 of 32 patients (90.6%). The ability of mNGS to identify microbial nucleic acid fragments without requiring viable organisms may partially explain its improved performance in slow-growing or metabolically inactive pathogens. Nevertheless, three tuberculosis cases were not detected by mNGS but were subsequently confirmed by histopathological findings, emphasizing that negative mNGS results should always be interpreted within the broader clinical context.

Importantly, mNGS expanded the etiological landscape of SI by revealing polymicrobial infections and uncommon pathogens, including *Coxiella burnetii*, *Talaromyces marneffei*, *Aspergillus fumigatus*, and oral microbiota-derived organisms (Yang et al., 2023; Kilinc et al., 2024). These findings are clinically relevant because atypical infections may closely mimic malignant or inflammatory disorders on imaging, resulting in diagnostic uncertainty and delayed pathogen-directed therapy. Representative cases are presented in **Supplementary Figures 4–S8**, highlighting the incremental diagnostic value of integrating radiological, histopathological, and microbiological findings in complex spinal infections. Nevertheless, detection of microbial nucleic acids alone does not necessarily indicate active infection, particularly in low-biomass specimens (Alrasheed et al., 2026). Therefore, integration with clinical presentation, pathological findings, and treatment response remains essential.

Prior antimicrobial exposure and sampling limitations represent important challenges in the diagnosis of SI, as they may reduce the yield of conventional microbiological methods. In our cohort, mNGS maintained robust sensitivity across antibiotic exposure status and different sampling approaches, including percutaneous biopsy, surgical specimens, and various tissue types, supporting its potential value when conventional pathogen recovery is compromised. These findings also suggest the feasibility of minimally invasive sampling strategies, particularly for patients with increased surgical risk (Chen et al., 2025). However, given the retrospective design and the limited availability of detailed information regarding antibiotic regimens, timing before sampling, and specimen acquisition procedures, residual confounding cannot be excluded (Yin et al., 2025). Future prospective studies with standardized assessment of antimicrobial exposure and sampling protocols are warranted to further define the optimal implementation of mNGS in SI diagnosis.

Although mNGS demonstrated high diagnostic sensitivity, its results should be interpreted within a comprehensive clinical context rather than considered definitive in isolation. In our cohort, three cases of spinal tuberculosis confirmed by histopathology and two culture-positive infections were not detected by mNGS, indicating that a negative result cannot reliably exclude SI when clinical suspicion remains high (Li et al., 2025). These false-negative findings and occasional taxonomic discrepancies among concordant-positive specimens may arise from factors such as low microbial biomass, nucleic acid degradation, or technical variability, underscoring the necessity of stringent quality control, contamination surveillance, and integration with complementary diagnostic modalities (Elbehiry and Abalkhail, 2025).

Several limitations should be acknowledged. First, this was a retrospective single-center study, and the observed pathogen distribution may partly reflect regional epidemiological characteristics and referral patterns, which may limit generalizability to other populations. Prospective multicenter validation studies are required to confirm the reproducibility of our findings. Second, although the multidisciplinary CRS used in this study provides a pragmatic framework for diagnostic adjudication, no universally accepted gold standard currently exists for SI. Third, targeted molecular assays such as GeneXpert MTB/RIF were not routinely available across the cohort, precluding systematic head-to-head comparison with mNGS. Prospective studies with standardized parallel testing are needed to further clarify their complementary roles in SI diagnosis. Fourth, the clinical implementation of mNGS remains constrained by economic cost, technical complexity, and the need for standardized bioinformatic interpretation. Although mNGS provides broad microbial detection capability, interpretation of resistance genes remains challenging because genotype–phenotype discordance is common, and conventional antimicrobial susceptibility testing remains necessary for therapeutic guidance. Finally, the current study focused primarily on diagnostic performance and short-term clinical implications. Larger prospective cohorts with longer follow-up are needed to determine the long-term clinical impact and cost-effectiveness of integrating mNGS into routine diagnostic algorithms for spinal infection.

## 5 Conclusion

In summary, mNGS provides a substantially higher diagnostic yield than conventional culture in spinal infections, particularly for fastidious and rare pathogens. Its performance remains robust despite prior antibiotic exposure and variations in sampling modality, thereby addressing key limitations of conventional microbiological testing. By facilitating earlier pathogen-directed therapy, mNGS may support antimicrobial stewardship and reduce unnecessary healthcare utilization. However, mNGS should be regarded as a complementary diagnostic modality rather than a stand-alone test, and its results should be interpreted within a multimodal framework that integrates clinical, imaging, and histopathological findings to optimize diagnostic accuracy and clinical management.

## Data Availability

The original contributions presented in the study are included in the article/**Supplementary Material**. Further inquiries can be directed to the corresponding author.
