# Supplementary material for: Integrating metagenomic next-generation sequencing into a multimodal diagnostic framework for spinal infection: enhancing etiological identification and clinical prediction
Source: Front Cell Infect Microbiol. 2026 Aug 24;16:1904634. doi: 10.3389/fcimb.2026.1904634 (PMC13547047; doi:10.3389/fcimb.2026.1904634)
Supplement: Supplementary file 1 [file Table1.docx]

**Supplementary Figures:**

**Supplementary Figure S1. Pathogen co-occurrence matrix and distribution between mNGS and conventional culture.**

**Supplementary Figure S2. Head-to-head comparison of ROC curves for five diagnostic modalities in common cases (N=190).**

*Note: AUC, area under the curve. This head-to-head comparison was performed on a subset of 190 common cases with complete data for all five modalities. The Integrated Clinical Model (incorporating clinical, imaging, and laboratory features) and the Conventional Biomarker Model (based on laboratory blood markers) were developed using L1-regularized logistic regression. 95% confidence intervals for AUC were estimated using the bootstrap method (n=1000).*

**Supplementary Figure S3.** **Regression coefficients of multimodal features selected for the integrated clinical model via L1-regularization.**

*Note: Features with non-zero coefficients were identified as the most significant predictors for spinal infection through the L1-regularized logistic regression (LASSO) process. Red bars represent positive coefficients, indicating risk factors (higher values or presence of the feature increases the likelihood of infection). Blue bars represent negative coefficients, indicating protective factors (higher values or presence of the feature decreases the likelihood of infection).*

*Abbreviations: ALB, albumin; CRP, C-reactive protein; DD, D-dimer; ESR, erythrocyte sedimentation rate.*

**Supplementary Figure S4. Diagnosis of Brucella spondylitis using multimodal evidence integration.** Preoperative radiography and CT (A–B) showed destructive changes at the L5–S1 level. MRI (C–G) demonstrated vertebral and paraspinal soft-tissue involvement suggestive of infectious spondylodiscitis, with interval improvement after treatment (H). Histopathological examination (I–J) revealed chronic inflammatory changes with necrotic bone fragments and fibrovascular proliferation. Despite negative culture results, mNGS detected *Brucella* spp., supporting the diagnosis of Brucella spondylitis when interpreted together with imaging and pathological findings.

**Supplementary Figure S5. Multimodal diagnostic assessment of culture-negative *Coxiella burnetii* spondylitis illustrating the incremental diagnostic value of mNGS.** Preoperative radiography and CT (A–C) demonstrated destructive vertebral lesions involving the thoracolumbar and lumbosacral spine. MRI (D–H) showed extensive spondylodiscitis with adjacent soft-tissue involvement. Histopathological examination (I–J) revealed nonspecific inflammatory changes without pathogen identification. Conventional cultures were negative, whereas mNGS detected *Coxiella burnetii*, enabling etiological diagnosis and highlighting the complementary role of mNGS in culture-negative spinal infections.

**Supplementary Figure S6. Multimodal diagnostic evaluation of Talaromyces marneffei spinal infection presenting as disseminated metastatic disease. A–C**, Radiography and CT demonstrated extensive multifocal osteolytic destruction with pathological fractures and associated soft-tissue masses. **D–H**, MRI revealed widespread vertebral and paravertebral involvement, producing imaging features highly suggestive of metastatic malignancy. **I–J**, Histopathological and immunohistochemical evaluation excluded neoplastic disease and demonstrated chronic granulomatous inflammation. Definitive etiological diagnosis was achieved through concordant detection of *Talaromyces marneffei* by both mNGS and conventional culture, highlighting the value of microbiological confirmation in diagnostically challenging spinal lesions that mimic malignancy.

**Supplementary Figure S7. Multimodal diagnostic evaluation of spinal tuberculosis. A–B**, Preoperative radiography and CT demonstrated destructive endplate changes and disc space narrowing at T6–T7. **C–G**, MRI revealed vertebral destruction, disc involvement, and paravertebral inflammatory extension, highly suggestive of tuberculous spondylitis. **H**, Postoperative radiograph after surgical treatment. **I–J**, Histopathological examination showed granulomatous inflammation with multinucleated giant cells and focal caseous necrosis. Acid-fast staining confirmed the presence of acid-fast bacilli. Although conventional culture remained negative, mNGS identified *Mycobacterium tuberculosis* complex, providing rapid etiological confirmation and illustrating the complementary role of mNGS in culture-negative spinal tuberculosis.

**Supplementary Figure S8. Multimodal diagnostic evaluation of concomitant spinal metastasis and tuberculosis. A–C**, Preoperative radiography and CT demonstrated extensive osteolytic destruction involving the L5 vertebra, posterior elements, sacrum, and bilateral iliac bones with pathological fracture. **D–H**, MRI revealed multifocal spinal and sacroiliac lesions with neural and soft-tissue involvement, findings highly suggestive of metastatic disease. **I–J**, Histopathological and immunohistochemical evaluation confirmed metastatic adenocarcinoma. Despite negative conventional cultures, mNGS detected *Mycobacterium tuberculosis* complex, providing evidence of concomitant spinal tuberculosis. The final diagnosis was established through integration of imaging, pathology, and microbiological findings, highlighting the value of mNGS in uncovering occult infectious etiologies in diagnostically complex spinal lesions.

**Supplementary Tables:**

**Supplementary Table S1. Comparison of mNGS-Detected ARGs and Phenotypic Resistance Rates Across Subgroups**

| Parameter / Group | Positive, n (%) | Negative, n (%) | Total, N | P Value |
| --- | --- | --- | --- | --- |
| mNGS ARG detection by phenotypic status |  |  |  | <.001 |
| Known phenotype | 25 (54.3) | 21 (45.7) | 46 |  |
| Unknown phenotype*** | 9 (10.5) | 77 (89.5) | 86 |  |
| mNGS ARG detection by prior antibiotic exposure |  |  |  | 0.824 |
| No prior exposure | 21 (25.3) | 62 (74.7) | 83 |  |
| Prior exposure | 13 (23.6) | 42 (76.4) | 55 |  |
| Phenotypic resistance by prior antibiotic exposure^#^ | Resistant, n (%) | Susceptible, n (%) | Total, N | 1.000 |
| No prior exposure | 21 (80.8) | 5 (19.2) | 26 |  |
| Prior exposure | 16 (80.0) | 4 (20.0) | 20 |  |

*Note: * Includes patients with culture-negative results or those without available phenotypic AST. ^#^ Analysis was restricted to the subset of patients with culture-positive results and available phenotypic AST data (n = 46). Abbreviations: ARG, antibiotic resistance gene; AST, antimicrobial susceptibility testing; mNGS, metagenomic next-generation sequencing.*

**Supplementary Table S2. Comparison of diagnostic performance among mNGS, conventional culture, and Histopathology.**

| Metrics | mNGS (n=208) | Culture (n=197) | Histopathology (n=201) |
| --- | --- | --- | --- |
| True positive (TP) | 127 | 53 | 101 |
| False negative (FN) | 11 | 79 | 30 |
| False positive (FP) | 10 | 1 | 7 |
| True negative (TN) | 60 | 64 | 63 |
| Sensitivity (%) *** | 92.03 (86.29–95.49) | 40.15(32.18–48.68) | 77.10 (69.19–83.46) |
| Specificity (%) *** | 85.71 (75.66–92.05) | 98.46 (91.79–99.73) | 90.00 (80.77–95.07) |
| PPV (%) *** | 92.70 (87.08–95.99) | 98.15 (90.23–99.67) | 93.52 (87.22–96.83) |
| NPV (%) *** | 84.51 (74.35–91.12) | 44.76 (36.85–52.94) | 67.74 (57.70–76.38) |
| Accuracy (%) *** | 89.90 (85.06–93.30) | 59.39 (52.42–66.01) | 81.59 (75.66–86.34) |
| Youden index | 0.777 | 0.386 | 0.671 |
| F1-score | 0.924 | 0.570 | 0.845 |

*Note: * Data are presented as percentages, with 95% confidence intervals in parentheses. mNGS, metagenomic next-generation sequencing; PPV, positive predictive value; NPV, negative predictive value.*

**Table S3. Consistency and head-to-head comparison between diagnostic methods.**

| Comparison (Method A vs. Method B) | N | Both (+) | A (+) / B (–) | A (–) / B (+) | Both (–) | Agreement (%) | Kappa coefficient | McNemar p-value |
| --- | --- | --- | --- | --- | --- | --- | --- | --- |
| mNGS vs. Culture | 197 | 51 | 80 | 3 | 63 | 57.87 | 0.27 | <0.001 |
| mNGS vs. Histopathology | 201 | 91 | 39 | 17 | 54 | 72.14 | 0.43 | 0.005 |
| Culture vs. Histopathology | 190 | 36 | 15 | 65 | 74 | 57.89 | 0.18 | <0.001 |

*Note: mNGS, metagenomic next-generation sequencing. Kappa coefficient was used to evaluate the strength of agreement (0.01–0.20 slight, 0.21–0.40 fair, 0.41–0.60 moderate, 0.61–0.80 substantial, and 0.81–1.00 almost perfect). McNemar’s test was used to compare the positive rates between the two methods.*

**Table S4. Diagnostic performance of mNGS, culture, and histopathology in the study cohort (N = 190).**

| **Metric** | **mNGS** | **Culture** | **Histopathology** |
| --- | --- | --- | --- |
| Sensitivity (%) *** | 91.20 (84.93-95.02) | 40.00 (31.83-48.76) | 76.00 (67.81-82.64) |
| Specificity (%) *** | 84.62 (73.94-91.42) | 98.46 (91.79-99.73) | 90.77 (81.29-95.70) |
| PPV (%) *** | 91.94 (85.79-95.56) | 98.04 (89.70-99.65) | 94.06 (87.64-97.25) |
| NPV (%) *** | 83.33 (72.57-90.43) | 46.04 (37.98-54.32) | 66.29 (55.98-75.26) |
| Accuracy (%) *** | 88.95 (83.69-92.66) | 60.00 (52.90-66.70) | 81.05 (74.89-85.99) |
| Youden index | 0.758 | 0.385 | 0.668 |
| F1-score | 0.916 | 0.568 | 0.841 |

*Note: * Data are presented as percentages, with 95% confidence intervals in parentheses. mNGS, metagenomic next-generation sequencing; PPV, positive predictive value; NPV, negative predictive value. All methods were performed on the same set of 190 clinical samples.*

**Table S5. Consistency and head-to-head comparison of diagnostic methods (N=190).**

| Comparison (Method A vs. Method B) | Both (+) | A (+) / B (–) | A (–) / B (+) | Both (–) | Agreement (%) | Kappa coefficient | McNemar p-value |
| --- | --- | --- | --- | --- | --- | --- | --- |
| mNGS vs. Culture | 48 | 76 | 3 | 63 | 58.42 | 0.27 | <0.001 |
| mNGS vs. Histopathology | 85 | 39 | 16 | 50 | 71.05 | 0.41 | 0.003 |
| Culture vs. Histopathology | 36 | 15 | 65 | 74 | 57.89 | 0.18 | <0.001 |

*Note: mNGS, metagenomic next-generation sequencing. Both (+) and Both (–) represent the number of samples where both methods yielded positive or negative results, respectively. A (+) / B (–) indicates Method A was positive while Method B was negative. Kappa coefficient was used to evaluate the strength of agreement. McNemar’s test was used to compare the difference in positive detection rates between the two methods.*

**Supplementary Table S6. Comparison of diagnostic accuracy and area under the curve (AUC) among different modalities.**

| **Method** | **Total cases(N)** | **CRS Positive(n)** | **CRS Negative(n)** | **AUC** | **95% CI** |
| --- | --- | --- | --- | --- | --- |
| Integrated clinical model | 208 | 138 | 70 | 0.916 | 0.870-0.952 |
| Conventional Biomarker Model | 208 | 138 | 70 | 0.742 | 0.673-0.808 |
| mNGS | 208 | 138 | 70 | 0.889 | 0.840-0.934 |
| Culture | 197 | 132 | 65 | 0.693 | 0.648-0.735 |
| Histopathology | 201 | 131 | 70 | 0.836 | 0.787-0.884 |

*Note: CRS, Composite Reference Standard (the final clinical diagnosis); AUC, area under the curve; CI, confidence interval; mNGS, metagenomic next-generation sequencing. The Integrated clinical model and the Conventional Biomarker Model were constructed using L1-regularized logistic regression. The 95% CI for AUC was calculated using the bootstrap method (n=1000).*

**Supplementary Table S7. Head-to-head comparison of diagnostic performance among five modalities in common cases (N=190).**

| Method | Total cases (N) | CRS Positive (n) | CRS Negative (n) | AUC | 95% CI |
| --- | --- | --- | --- | --- | --- |
| Integrated Clinical Model | 190 | 125 | 65 | 0.911 | 0.859-0.952 |
| Conventional Biomarker Model | 190 | 125 | 65 | 0.745 | 0.670-0.813 |
| mNGS | 190 | 125 | 65 | 0.879 | 0.828-0.927 |
| Culture | 190 | 125 | 65 | 0.692 | 0.649-0.741 |
| Histopathology | 190 | 125 | 65 | 0.834 | 0.780-0.885 |

*Note: CRS, Composite Reference Standard (the final clinical diagnosis); AUC, area under the curve; CI, confidence interval; mNGS, metagenomic next-generation sequencing. This analysis was restricted to common cases (N=190) with complete data for all modalities to eliminate selection bias. The Integrated Clinical Model and Conventional Biomarker Model were constructed using L1-regularized logistic regression. The 95% CI for AUC was estimated using the bootstrap method (n=1000).*

**Supplementary Table S8. Regression coefficients of multimodal features selected for the integrated clinical model via L1-regularization.**

| Feature (Variable) | Coefficient |
| --- | --- |
| **Imaging Characteristics:** |  |
| Disc invasion (Yes) | 1.079 |
| Paraspinal inflammation (Yes) | 0.8 |
| Vertebral body invasion (Yes) | 0.056 |
| Compressive lesion (Yes) | 0.056 |
| Paraspinal inflammation (No) | -0.427 |
| Disc invasion (No) | -0.704 |
| Involved segments (Single-segment) | -2.043 |
| **Laboratory Findings:** |  |
| CRP (mg/L) | 0.595 |
| ESR (mm/h) | 0.453 |
| Albumin (g/L) | -0.233 |
| Monocyte count | -0.233 |
| D-dimer (ng/mL) | -0.289 |
| **Clinical Characteristics:** |  |
| Osteoporosis (No) | 0.34 |
| Age (years) | -0.026 |
| Osteoporosis (Yes) | -0.105 |

*Note: Features with non-zero coefficients were identified as the most significant predictors for spinal infection through the L1-regularized logistic regression (LASSO) process. Red bars represent positive coefficients, indicating risk factors (higher values or presence of the feature increases the likelihood of infection). Blue bars represent negative coefficients, indicating protective factors (higher values or presence of the feature decreases the likelihood of infection). Abbreviations: ALB, albumin; CRP, C-reactive protein; DD, D-dimer; ESR, erythrocyte sedimentation rate.*

**Supplementary Table S9. Clinical Outcomes and Treatment Management of the Study Population (N = 138)**

| Variable | Value |
| --- | --- |
| Hospitalization duration, days, median (IQR) | 18 (13-25.3) |
| Follow-up duration, days, median (IQR) | 73 (31-165.5) |
| Surgical Management, n (%) |  |
| Surgical intervention | 119 (86.2) |
| Conservative management | 19 (13.8) |
| Antimicrobial Treatment Management, n (%) |  |
| Adjustment based on pathogen identification | 112 (81.2) |
| Pathogen-directed therapy*** | 108 (78.3) |
| De-escalation of therapy | 4 (2.9) |
| No adjustment (Maintained initial regimen) | 26 (18.8) |
| Clinical Status at Discharge, n (%) |  |
| Clinical improvement | 134 (97.1) |
| Clinical worsening | 3 (2.2) |
| In-hospital mortality | 1 (0.7) |
| Final Follow-up Outcomes, n (%) |  |
| Improvement | 125 (90.6) |
| Recurrence | 4 (2.9) |
| mechanical failures | 2 (1.4) |
| Mortality | 7 (5.1) |

*Note: * Pathogen-directed therapy was defined as pathogen-specific treatment, including anti-tuberculosis, anti-Brucella, antifungal, or susceptibility-guided (targeted) antibacterial regimens. Abbreviations: IQR, interquartile range.*

**Supplementary Table S10. Summary of antimicrobial management according to pathogen categories in patients with spinal infection**

| Etiological category | Representative pathogens | Typical antimicrobial regimen |
| --- | --- | --- |
| Pyogenic infection | *Staphylococcus aureus, Escherichia coli, Klebsiella pneumoniae* | Targeted antibacterial therapy guided by culture, mNGS findings, and antimicrobial susceptibility testing (e.g., vancomycin, linezolid, cefoperazone, levofloxacin, or piperacillin–tazobactam) |
| Tuberculous infection | *Mycobacterium tuberculosis complex* | Standard four-drug anti-tuberculosis regimen |
| Brucellar infection | *Brucella spp.* | Doxycycline plus rifampicin–based combination therapy |
| Fungal infection | *Aspergillus fumigatus, Talaromyces marneffei* | Species-directed antifungal therapy (e.g., voriconazole or posaconazole-based regimens) |
